# Long-Range Effects in Topologically Defective Arm-Chair Graphene Nanoribbons

**DOI:** 10.3390/nano14090778

**Published:** 2024-04-30

**Authors:** Enrique Louis, Guillermo Chiappe, José A. Vergés, Emilio San-Fabián

**Affiliations:** 1 Departamento de Física Aplicada, Instituto Universitario de Materiales de Alicante (IUMA), Universidad de Alicante, 03080 Alicante, Spain; enrique.louis@ua.es; 2 Departamento de Teoría y Simulación de Materiales, Instituto de Ciencia de Materiales de Madrid (CSIC), Cantoblanco, 28049 Madrid, Spain; jav@icmm.csic.es; 3 Departamento de Química Física, Instituto Universitario de Materiales de Alicante (IUMA), Universidad de Alicante, 03080 Alicante, Spain

**Keywords:** graphene, nano-ribbons, long-range effects

## Abstract

The electronic structure of 7/9-AGNR superlattices with up to eight unit cells has been studied by means of state-of-the-art Density Functional Theory (DFT) and also by two model Hamiltonians, the first one including only local interactions (Hubbard model, Hu) while the second one is extended to allow long-range Coulomb interactions (Pariser, Parr and Pople model, PPP). Both are solved within mean field approximation. At this approximation level, our calculations show that 7/9 interfaces are better described by spin non-polarized solutions than by spin-polarized wavefunctions. Consequently, both Hu and PPP Hamiltonians lead to electronic structures characterized by a gap at the Fermi level that diminishes as the size of the system increases. DFT results show similar trends although a detailed analysis of the density of states around the Fermi level shows quantitative differences with both Hu and PPP models. Before improving model Hamiltonians, we interpret the electronic structure obtained by DFT in terms of bands of topological states: topological states localized at the system edges and extended bulk topological states that interact between them due to the long-range Coulomb terms of Hamiltonian. After careful analysis of the interaction among topological states, we find that the discrepancy between ab initio and model Hamiltonians can be resolved considering a screened long-range interaction that is implemented by adding an exponential cutoff to the interaction term of the PPP model. In this way, an adjusted cutoff distance λ=2 allows a good recovery of DFT results. In view of this, we conclude that the correct description of the density of states around the Fermi level (Dirac point) needs the inclusion of long-range interactions well beyond the Hubbard model but not completely unscreened as is the case for the PPP model.

## 1. Introduction

Since its rediscovery in 2004 and isolation and investigation by a Manchester team (very particularly by A.K. Geim and K.S. Novoselov, see Refs. [1,2]) graphene is offering physicists and chemists an ever larger and richer field to test the body of knowledge developed by researchers during the last one hundred years. Graphene, besides the expectations regarding technological applications, is defying the community of condensed matter physicists up to limits by no means anticipated [3]. Almost all experimental and theoretical tools developed in that period are finding a place in the flourishing field of graphene. Starting from its Dirac character at energies near the Fermi level, the novel superconductivity observed in twisted bi-layers [3,4] along with zero-width bands which suggests the high relevance of electron–electron interaction in defining what points to be non-BCS superconductivity, as in high-temperature superconductors.

Changes in the chemical potential and a rearrangement of the low-energy excitations at each integer filling of the Moiré flat bands are being identified. These spectroscopic features are a direct consequence of Coulomb interactions, which split the degenerate flat bands into Hubbard sub-bands. The cascade of transitions reported up to now characterizes the correlated high-temperature parent phase from which various insulating and superconducting ground-state phases emerge at low temperatures in MATBG [5,6]. After the discovery of these effects in twisted layered samples, experimental and theoretical efforts are being addressed to characterize the samples trying to find out how this behavior depends on filling, the number of layers and the twisting angle, the three variables that seem to be crucial. The story, however, seems to be at the beginning, as recent experimental work clearly indicates that there is no need to twist the layered sample [3] to produce the superconductivity and other effects similar to those observed in twisted samples.

**Figure 1:**
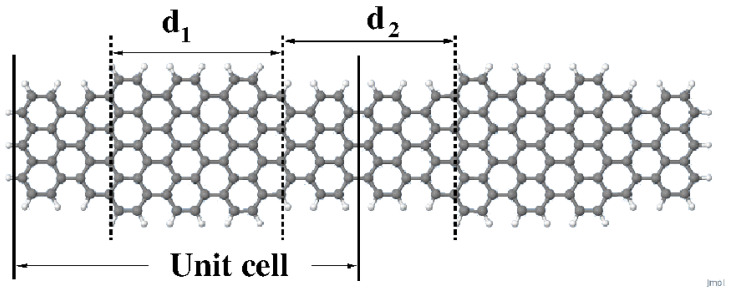
Schematic representation of the 7/9-AGNR with two unit cells (we call this cluster C2).

The first evidence of topological states in junctions of arm-chair graphene nanoribbons (AGNR) of different width grown on Au(111) surfaces were reported in 2018 [7,8]. It was also shown that these states can be described following a tight-binding model proposed by Su, Schrieffer and Heeger (SSH) [8,9,10]. Combining theoretical analyses with already developed bottom-up techniques they were able to produce and characterize graphene samples with and without topological defects. Taking as a unit cell the junction 7/9-AGNR, they studied various configurations ranging from an isolated 7–9 junction up to an infinite linear arrangement of 7–9 units (See Figure 1 as an example containing four 7–9 junctions). They chose the 7–9 unit because one of the two possible ways to join the two ribbons (just the one shown in Figure 1) hosts an electronic topological state. Analysis of the effect of interactions on these 7/9-AGNR superlattices deposited on Au(111) surfaces was also conducted in the context of the Hu model [11].

In this work, we focus on the effects of long-range electronic interactions on the coupling between topological states raised on free-standing 7/9-AGNRs superlattices. More concretely, we consider finite 7/9-AGNR superlattices (as the own shown in Figure 1) that are known to show topological defects at each 7–9 interface. Extensive calculations of the electronic structure of 7/9-AGNR superlattices with up to eight unit cells using PPP [12,13], Hu [14] (specifically, just to check the local limit of the previous model) and B3LYP DFT [15,16,17,18] schemes have been undertaken. Although the band analysis can be performed from a periodic system calculation, we work with finite systems, which facilitate the analysis of molecular orbitals and edge effects. From the comparison of the calculated density of states (DOS) around the Fermi level, the need to improve the long-range interactions of the approximate PPP model Hamiltonian arises. We have been able to propose a model of topological first-neighbors interacting states that correctly describes the DOS in the neighborhood of the Fermi level. Using this simplified model, the electronic structure of much larger systems can be precisely obtained.

## 2. Computational Methods

The Hamiltonians we shall use are the B3LYP ab initio DFT and the Pariser, Parr and Pople (PPP). Both methods incorporate short and long-range electron–electron interaction and were handled within the restricted or unrestricted approximations that have been intensively applied to investigate the electronic structure of polycyclic aromatic hydrocarbon (PAH) [19].

DFT calculations were carried out using the B3LYP exchange-correlation functional [15,16,17,18] and the basis set 6-31G* [20,21], using the Gaussian-16 computational package [22]. All geometries have been optimized at B3LYP/6-31G* level. Polarized solutions are obtained using the unrestricted approximation of both methods, which provide solutions with $S_{z}\;=\;0$ and <S>>0. In place, non-polarized solutions are calculated using the restricted approximation of both methods which provides solutions with $S_{z}\;=\;0$ and S=0. The densities of states (DOS) were calculated either by means of Green functions (PPP) or with Gaussian-16 and with the help of GaussSum [23] and Multiwfn [24] programs. We use an artificial Gaussian broadening function, a half-width of 0.05 eV and the Mülliken method to calculate the partial DOS (PDOS).

The PPP model Hamiltonian contains, besides the standard kinetic energy, both local on-site and long-range Coulomb interactions and a single π orbital per carbon atom. The non-interacting term incorporates two standard parameters, the orbital energy $\epsilon_{0}$ and the hopping between nearest-neighbor pairs $t_{ij}$, namely,

(1) $${\hat{H}}_{0}=\epsilon_{0}\sum_{i=1,N;\sigma }{\hat{c}}_{i\sigma }^{†}{\hat{c}}_{i\sigma }+\sum_{<ij>;\sigma }t_{ij}{\hat{c}}_{i\sigma }^{†}{\hat{c}}_{j\sigma }\;,$$

where the operator ${\hat{c}}_{i\sigma }^{†}$ creates an electron at site *i* with spin σ, *N* is the number of orbitals and $t_{ij}$ is the hopping between nearest-neighbor pairs <ij>.

In cases where the distance $d_{ij}$ between nearest neighbor pairs significantly deviates from the standard value, $d_{0}=1.41$ Å, due, for instance, to defects or impurities, the hopping parameter may be scaled using the following scaling law adequate for π orbital [25] namely,

(2) $$t_{ij}={\left(\frac{d_{0}}{d_{ij}}\right)}^{3}.$$

Regarding the values of the model parameters, we use the well-tested set reported in Ref. [26].

Within the Hartree–Fock approximation (HF), the interacting term of the PPP Hamiltonian is approximated by:

$${\hat{H}}_{I-PPP}^{HF}=U\sum_{i=1,N;\sigma }\left({\hat{n}}_{i\sigma }\langle {\hat{n}}_{i\bar{\sigma }}\rangle -\frac{1}{2}\langle {\hat{n}}_{i\sigma }\rangle \langle {\hat{n}}_{i\bar{\sigma }}\rangle \right)$$

$$\;-\frac{1}{2}\sum_{i\neq j}V_{ij}\left(\langle {\hat{n}}_{i}\rangle \langle {\hat{n}}_{j}\rangle -\sum_{\sigma }\langle {\hat{c}}_{i\sigma }^{†}{\hat{c}}_{j\sigma }\rangle \langle {\hat{c}}_{i\sigma }^{†}{\hat{c}}_{j\sigma }\rangle -1\right)$$

(3) $$+\sum_{i\neq j}V_{ij}\left({\hat{n}}_{i}\langle {\hat{n}}_{j}\rangle -\sum_{\sigma }{\hat{c}}_{i\sigma }^{†}{\hat{c}}_{j\sigma }\langle {\hat{c}}_{i\sigma }^{†}{\hat{c}}_{j\sigma }\rangle \right)\;.$$

Here, ${\hat{n}}_{i}\;=\;\sum_{\sigma }{\hat{n}}_{i\sigma }$ and ${\hat{n}}_{i\sigma }$ is the occupation number in the site *i* with spin σ. The first line of this equation is the HF version of the Hubbard Hamiltonian (Hu), which only retains local interactions. It is interesting to note the presence of non-diagonal terms in the third parenthesis of the last equation. These terms introduce frustration in non-frustrated lattices. This is surely the reason why the staggered polarization in the polarized configuration is always smaller in the PPP than in the Hu model.

In incorporating the interaction $V_{ij}$ in the PPP model, one may choose the unscreened Coulomb interaction [27] although it is a common practice to use interpolating formulae. In the case of PAHs, that proposed by Ohno [28] has wide acceptance. We use that formula, modified to incorporate a parameter λ that allows us to control the extent of the long-range interactions. When $\frac{1}{\lambda }\;\to \;0$ the unscreened Coulomb interaction is recovered:

(4) $$V_{ij}=Ue^{-\frac{d_{ij}}{\lambda }}{\left[1+{\left(\frac{Ud_{ij}}{e^{2}}\right)}^{2}\right]}^{-1/2}\;,$$

where U=8.36 eV, $d_{ij}$ is the distance between *i* and *j* atoms, in Angstroms and *e* is the electron charge. The parameter λ will be fixed in order to better reproduce ab initio DFT results with the PPP model.

Supercells containing 2, 4, 6 and 8 unit cells were in most cases used to illustrate our findings (see Figure 1 for a schematic representation of the unit cell). Other geometries were occasionally used to reinforce a given argument. Geometries were optimized for non-polarized wavefunctions at the B3LYP/6-31G* level. Optimized geometries do not differ much from the bulk geometry of graphene and depend on the number of cells considered, as seen in Table 1, where $d_{1}$ and $d_{2}$ are the lengths of the ribbons having widths 7 and 9, respectively.

## 3. Results and Discussion

### 3.1. Checking Computational Approach

To guarantee the robustness of our results, four combinations of exchange-correlation functional/basis sets were investigated. Specifically, an alternative PBEPBE [29] functional and an extended 6-311G* [30] basis set were considered. Results are shown in Table 2.

**Figure 2:**
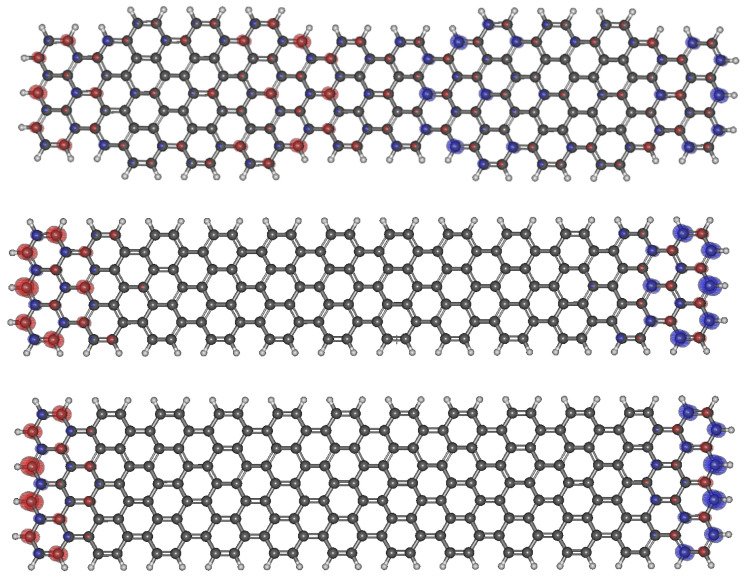
Spin density of th e polarized solutions of 7/9, 7/7 and 9/9 AGNRs.

In the context of the present work, the most interesting property is the forbidden gap. The results for four combinations of functional/basis sets are all within the range 0.344–0.440 eV. We guess that other sources of errors are more important than this difference. Therefore, from this point on, the DFT calculations carried out in this work have been conducted with the B3LYP/6-31G* choice. The relevance of solutions with polarization versus those non-polarized, as well as the variation of the gap when the 7/9 interfaces are formed or not has been analyzed for the case of two unit cells and their equivalents without interfaces (7/7 and 9/9). Their spin densities are shown in Figure 2. Table 3 summarizes our results.

It is clear that our calculations indicate that polarized solutions are more stable (see Table 3) but the difference in stability with the non-polarized solutions is much smaller when topological defects (7/9 interfaces) are present. This suggests that topological defects tend to stabilize these solutions even at the DFT level. Also, polarized solutions, in general, exhibit larger gaps due to an overestimation of exchange in the mean field solutions.

Therefore, hereafter as the number of cells increases, we shall focus on non-polarized DFT solutions, which preserve S=0 and provide a more adequate description of energies next to the Fermi level. Likewise, the polarized defect-free solutions show a much larger gap than solutions for the 7/9-AGNR system. Meanwhile, the non-polarized solutions for 7/7 and 9/9 hardly show any gap; 7/9-AGNR shows a moderate gap (see Table 3). However, we will show below that this gap closes when the size is increased.

### 3.2. Characterizing Edge and Topological States

**Figure 3:**
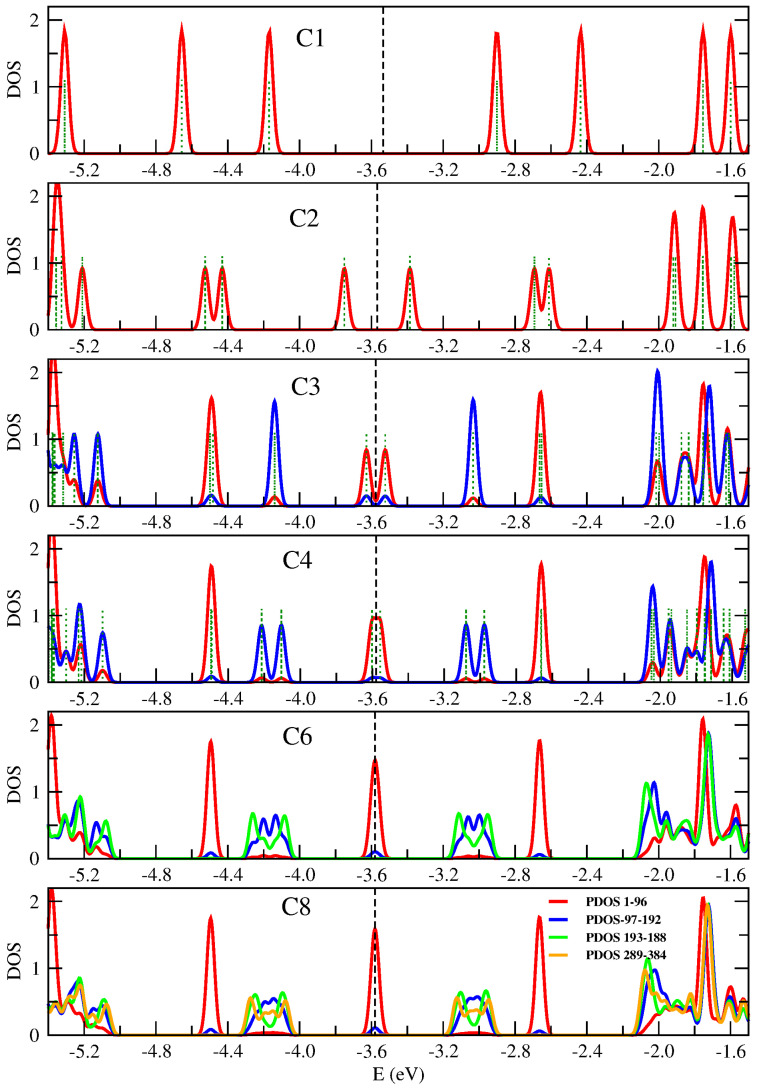
Spatially decomposed density of states of 7/9-AGNR superlattices with one to eight unit cells (C1–C8) calculated by means of B3LYP/6-31G* method. Partial densities of states (PDOS) were obtained by adding up the local density of states on symmetrical Cs in contiguous layers of 96 atoms (1–96, …, 289–384). For C1 to C4 the eigenvalues are shown with a dark green dotted line. Results are given in the energy region where topological states show up, that is, between −4.8 and −2.4 eV (the Fermi level is approximately at −3.6 eV).

In order to study the effect of topological states rising up in the frontiers of 7/9-AGNR superlattices, we calculate the partial density of states (PDOS) in the vicinity of the Fermi level spatially discriminating the unit cells of the sample. Summing up all these PDOS gives the total DOS of the system. Figure 3 shows PDOS for superstructures C1 to C8. Note, that C1, the unit cell, includes 96 atoms. Partial densities of states for different C’s were obtained by adding up the local density of states corresponding to each unit cell (a layer with 96 atoms) and its symmetrical relative to the center of the sample. Therefore, the red curves in Figure 3 give the PDOS for the first cell in the sample and it is symmetrical (the last unit cell). Blue curves give the PDOS for the second cell and it is symmetrical. Green curves for the third cell and it is symmetrical and the orange curve for the fourth cell and it is symmetrical.

We have three localized states corresponding to topological edge states, one just at, one below and one above the Fermi level (red lines in the figure), which would be modeled as a three-initially degenerated level system at the Fermi level with an effective coupling between them that breaks the degeneracy. In addition, we have two broader bands, below and above the Fermi level, corresponding to topological bulk states. No gap is present for the larger systems.

It is worth discussing in detail how the DOS around the Fermi level evolves with size. For this purpose, we will use a tight-binding model consisting of localized states around 7/9 frontiers (topological states) plus one more localized state at each of the system ends. The existence of localized states at the end of some GNR segments and at the junction between two GNRs was already proposed based on a topological analysis [31,32]. We start with the smallest superstructure C1. Although it is a special case because the ends of the system are relatively close to each other, it is useful to describe it within the kinds of ideas that will later be used to analyze larger sizes. A unit cell contains two 7/9 frontiers and two edges. There are four states, two coming from the edge states, and two from states localized at the frontiers, which are split by interactions amongst them due to the long-range term of the Coulomb interaction, raising the four peaks seen in the first panel of Figure 3. Two peaks correspond to states that are localized at edges (at Fermi level ±1.1 eV) and two more that are localized around the 7/9 interfaces and appear closer to the Fermi level.

**Figure 4:**
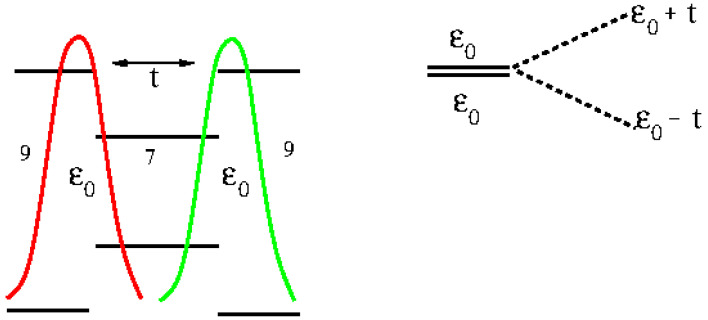
Schematic representation of topological bulk states localized at the internal boundaries of a unit cell in the bulk. When further cells are added to the system, an extended chain with two levels per unit cell is obtained.

**Figure 5:**
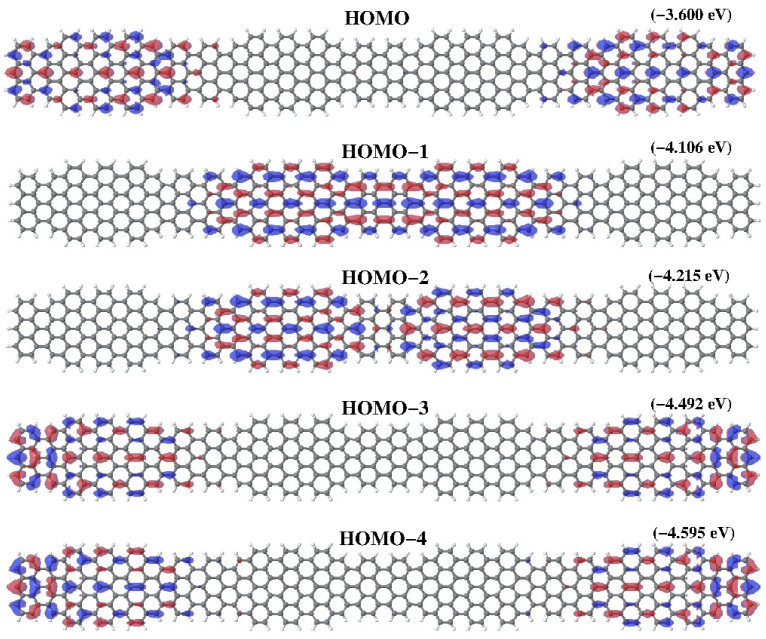
Higher occupied molecular orbitals obtained for a C4 superlattice containing four unit cells. First, fourth and fifth molecular orbitals are border states produced by the coupling of the edge state with the two topological states at cluster ends, while second and third molecular orbitals appear from the coupling of pure topological states at the central units. In brackets, their energy.

Going back to the results of Figure 3, we apply the same scheme to analyze C2. This superstructure contains two unit cells, each one with two topological states, and each one now brings only one edge state. So, we have six states that split into pairs below and above the Fermi level. The pair of peaks far away from the Fermi level correspond to states more localized at the edges of the sample so we call them edge states. Next, there are two pairs of peaks, the first corresponding to states more localized at the first 7/9 frontier and the pair of states closer to the Fermi level correspond to states localized around the two internal frontiers of C2. The structure of peaks (red peaks in all panels) that rise from it will stay for larger samples. In larger samples, it represents the DOS corresponding to the edge units of the sample. However, the edge units will become less interacting as the sample becomes larger, and then the peaks associated with it evolve to a three peaks structure, doubly degenerated, one at the Fermi level and two additional peaks, one below and another above it, like two isolated three sites open chain. We suggest that these peaks correspond to topological edge states. From C3 to C8, the peak features around −4.5 and −2.6 eV can be separated in pieces by the location of the states in the sample following the same ideas. For C3 we have three unit cells. Therefore, we will see two edge units, that provide the red peaks. In addition, we have two topological states in the central unit, which gives the blue peaks. These two states are split by interactions between them and with edge units. We call these states topological bulk states (See Figure 4). For C4, the peaks coming from the edge units close to the Fermi level are already merged in only one peak at the Fermi level because of the weakness of the interaction they suffer. Therefore, it will be so for larger strips. Now we have four topological bulk states at the two central units. These four topological bulk states can be described as a two-site chain, and each site represents a unit with two interacting levels. This corresponds to the four split blue peaks around the Fermi level. The characteristics of the molecular orbitals close to the Fermi level will help present an analysis of the local densities of states. DFT results for the five HOMO of higher energy in C4 are depicted in Figure 5. All of them contribute to different peaks of the PDOS. The fourth (*HOMO-3*) and fifth state (*HOMO-4*) are clearly edge states that correspond to the red peak in Figure 3 with energy around −4.5. The first state (*HOMO-0*) corresponds also to an edge state with energy around the Fermi level (−3.6, red peak). The second and third states (*HOMO-1* and *HOMO-2*) correspond to blue peaks with energy around −4.2, which are bulk states. For C6 and C8 the analysis follows the same way. The edge states, red lines, remain almost unchanged in C6 (C8). Now, we have more central units that can be modeled like a tight-binding chain with two-level sites that provide the peaks of blue, green and orange. The results for C8 compare qualitatively well with results shown in [7] for a similar superlattice grown on gold. Nevertheless, in our free-standing system, bands are broader and localized peaks become separated.

### 3.3. Tight-Binding Model for Localized States

**Figure 6:**
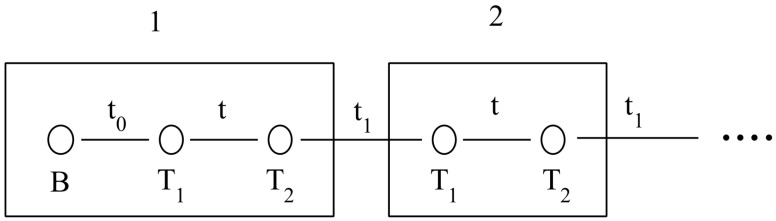
Tight-binding model for topological states. 1 is the unit cell at the borders of the sample. It contains three levels, one due to the border (B) and two due to the 7/9 frontiers $\left(T_{1}\right)$ and $\left(T_{2}\right)$. 2 is a bulk cell that contains only $\left(T_{1}\right)$ and $\left(T_{2}\right)$ states. $t_{0}$, *t* and $t_{1}$ are the couplings between them.

**Figure 7:**
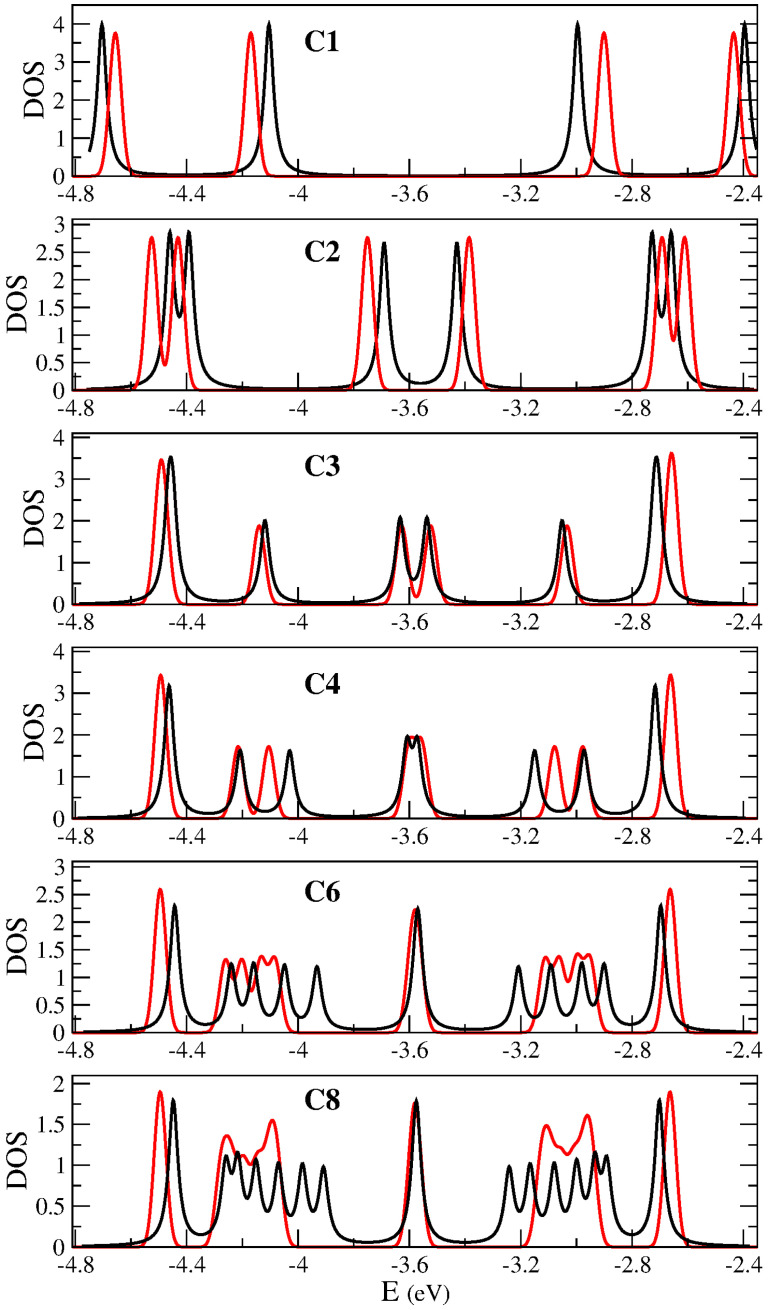
Black lines show the total DOS for superlattices C1 to C8, calculated using the topological tight-binding model with $t_{0}=0.7$ eV, t=0.6 eV and $t_{1}=0.3$ eV. Red lines show the total DOS obtained for the same systems using DFT.

**Figure 8:**
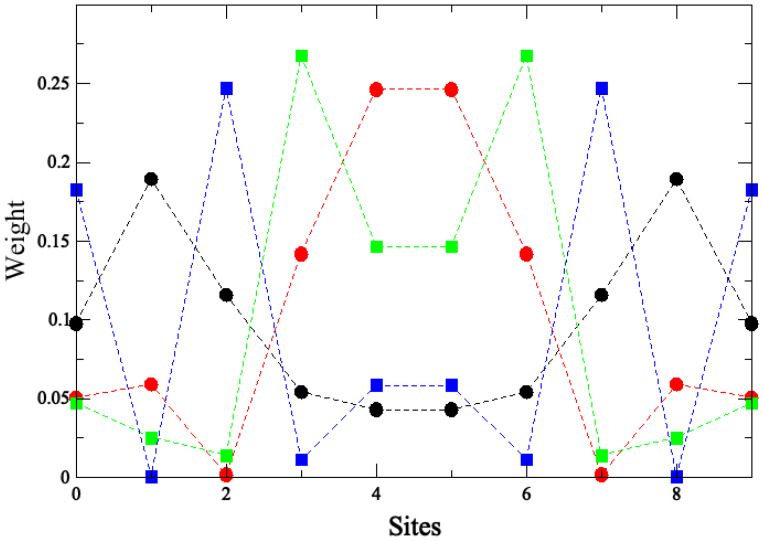
Molecular states of the tight binding model of topological states for C4. It has ten states. $t_{0}=0.7$ eV, t=0.6 eV and $t_{1}=0.3$ eV. See explanation in Section 3.3.

A tight-binding scheme has been employed in the previous subsection to analyze ab initio DFT results. Here, the whole simple model describing topological states will be given, following the idea of SSH model [8,9]. In our model, superlattices present two states $T_{1}$ and $T_{2}$ in bulk cells (see Figure 6) and three states *B*, $T_{1}$ and $T_{2}$ at both system ends. The energy of all these states is the same $\epsilon_{0}\;=\;E_{F}$ and they interact only with their first neighbors (in fact, as stated below, the correct description of DOS using PPP requires the screening of the Coulomb interaction up to distances of the order of a lattice parameter). In order to reproduce the results of Figure 3, we take $t_{0}=0.7$ eV, t=0.6eV and $t_{1}=0.3$ eV. Figure 7 shows results for the total DOS corresponding to C2−C8 superlattices as it is obtained using this tight-binding description of localized states. Also, we plot the weights of the molecular orbitals in the tight binding model in order to compare them with results shown in Figure 5 for the C4 superlattice (See Figure 8). Black circles and blue squares correspond to states in the border with energies around −4.5 and −3.6 like *HOMO-4* or *HOMO-3* and *HOMO-0* in Figure 5. Red circles and green squares correspond to states in the bulk with energies around −4.1 and −4.2, like *HOMO-2* and *HOMO-1* in Figure 5.

**Figure 9:**
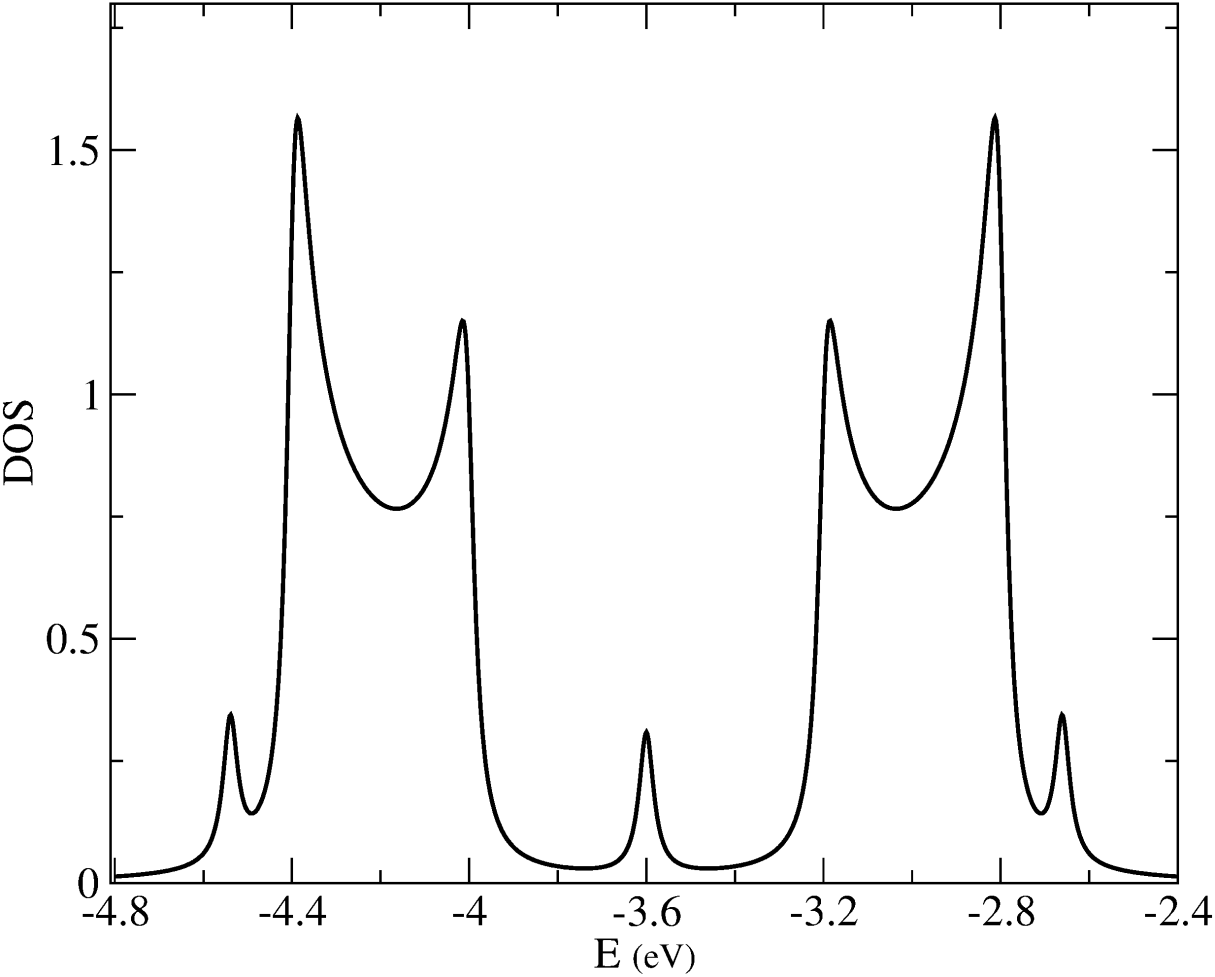
Total DOS for a C54 superlattice, calculated using the topological tight-binding model with $t_{0}=0.7$ eV, t=0.6 eV and $t_{1}=0.3$ eV.

The limit of DOS for larger superlattices can be obtained using the topological tight-binding model. Figure 9 shows the DOS for a C54 superlattice, where the bands of topological states are clearly developed.

### 3.4. A Screened PPP Model

**Figure 10:**
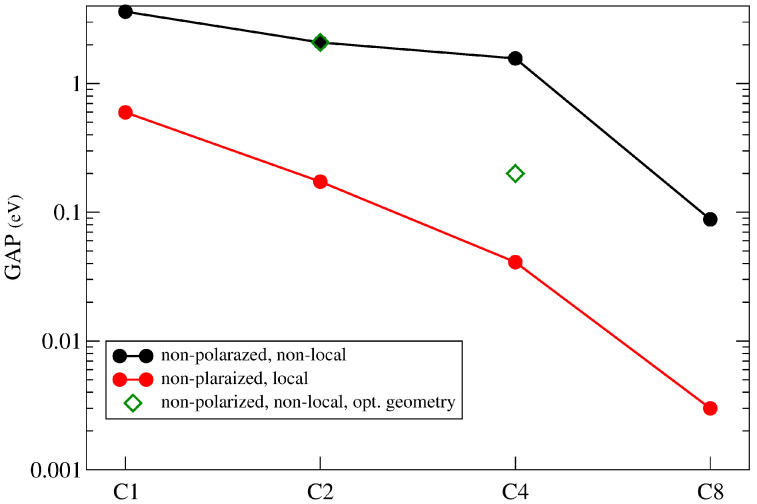
Forbidden gap of 7/9-AGNR superlattice vs the number of unit cells C1, C2, C4, C8. The results correspond to unrestricted non-polarized solutions of the PPP Hamiltonian including or not the long-range interaction (black and red circles, respectively) using a standard non-optimized geometry for the superlattice. Some results obtained by using the DFT-optimized geometry of previous calculations (green rhombus) are also shown.

It is interesting to wonder if this picture can be supported by the PPP model, mainly concerning the electronic structure near to the Fermi level. Table 4 and Figure 10 shows the forbidden gap obtained with PPP. We include, for completeness, results for polarized solutions also, but we will focus finally on non-polarized solutions.

Let us compare the Hu and PPP model Hamiltonians. The main features of our results are as follows: (i) The Hu model and non-polarized solutions lead to small gaps for any size, a result which is in line with DFT results. (ii) The PPP model produces slightly larger gaps, but they also tend to zero as size of the system increases. (iii) Polarized solutions show a large gap in any case.

**Figure 11:**
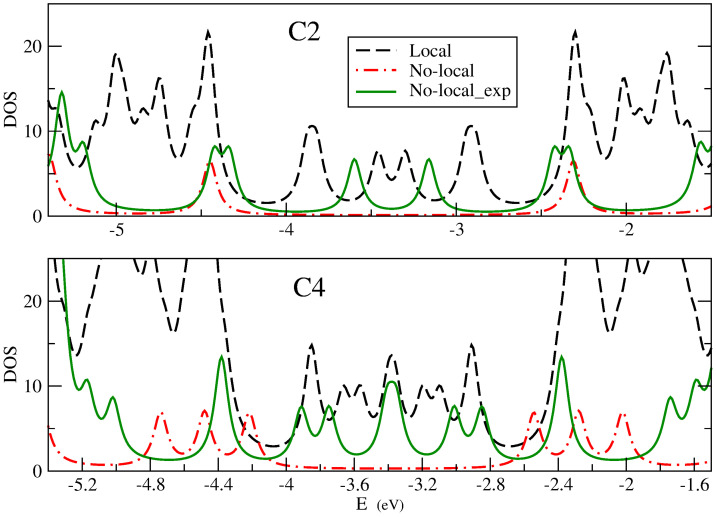
TDOS of 7/9-AGNR superlattice with 2 and 4 unit cells (C2 and C4), described by the Hu model (black line), the PPP model (red line) and the exponentially screened PPP model (green line).

In principle, both Hu or PPP models lead to zero gaps for non-polarized solutions as the size is increased. However, as Figure 11 shows, important differences relative to DFT results arise. The Figure shows the variation of TDOS using PPP (red line) and Hu (black line) models for C2 and C4 structures. Although the Hu interaction gives the DFT result correctly at the Fermi level (a small gap for C2 and a peak for C4) around the Fermi level the description is not that similar because it has a larger density of states than DFT. For the PPP model, we obtain a much larger gap. Both facts are due to the incorrect representation of the interaction between topological states coming from both models. Local interaction underestimates it and non-local interaction overestimates it. To improve these results, we use the $V_{i,j}$ screened potential, adjusting the λ factor. We have found that choosing λ=2 Å produces results that are qualitatively similar to DFT results (green line in the figure). This screened interaction potential seems to represent more accurately the physics of these strips. It is also consistent with the tight-binding model for topological states.

## 4. Final Remarks

The data presented in this work support the following conclusions and remarks:

(1) The combination of model Hamiltonian (Hu and PPP) calculations and ab initio DFT allows for the identification of the effective range of electronic interactions in free-standing 7/9-AGNR superlattices. In order to modulate the decay of the electron–electron interaction, an exponential cutoff has been used. The inclusion of the λ parameter is a crucial step for improving the agreement between PPP and DFT results, particularly concerning the description of DOS around the Fermi level. We find that λ=2 Å, a value that is somewhat larger than the parameter lattice, improves the resemblance between PPP and DFT DOS. Neither Hu nor unscreened long-range PPP models seem to be appropriate for the inclusion of interactions in these systems.
(2) We have given a plausible route for the evolution of the system density of states in going from the smallest C1 up to the largest superlattice C8. Localized states at 7/9 junctions evolve developing two kinds of states: three border states at each system end corresponding to three peaks in DOS at energies around −4.5, $E_{f}\;=\;-3.6$ and −2.5 (more precisely, they come from a combination of one pure border state and two topological states in the border unit cell) and bulk states that form two bands, one between −4.5 and $E_{f}$ and a symmetric second one that lies above $E_{f}$ (they are linear combinations of topological states originated at 7/9 junctions). All these features can be described by a tight-binding model of two levels per unit cell, except at the border unit cells where there are three states. The tight-binding model allows the precise calculation of the DOS of very large superlattices that are beyond the capability of ab initio methods.
(3) Our results for free-standing nanoribbons compare qualitatively well with previous results of similar systems deposited on Au (111) surfaces. Finite range interactions imply a renormalization of bandwidths and separations between peaks.
(4) We guess that the screened PPP model can be successfully employed in a large variety of geometric variations of defective free-standing nanoribbons allowing a quick exploration of new systems.

## Figures and Tables

**Table 1:** Geometrical structure of 7/9-AGNR superlattices with 2, 3, 4, 6 and 8 Cn)) unit cells calculated by means of DFT. In addition, results for the energies (ε) of HOMO and LUMO (in Hartrees) and the gap (in eV), are reported.

	C2: 192 C’s	C3: 288 C’s	C4: 384 C’s	C6: 576 C’s	C8: 768 C’s
$d_{1}$ (nm)	1.285	1.284	1.284	1.284	1.284
$d_{2}$ (nm)	1.295	1.293	1.293	1.293	1.293
$\varepsilon_{HOMO}$	−0.13783	−0.1333	−0.13234	−0.13198	−0.13187
$\varepsilon_{LUMO}$	−0.12441	−0.1294	−0.13065	−0.13112	−0.13126
Gap	0.365	0.107	0.046	0.023	0.017

**Table 2:** Several properties of 7/9-AGNR superlattice C2, calculated by means of DFT, using two different functionals and two Gaussian basis sets. All energies are in Hartrees, except the gap which is given in eV. See Figure 1 for $d_{1}$ and $d_{2}$ definitions.

	B3LYP/6-31G*	PBE/6-31G*	B3LYP/6-311G*	PBE/6-311G*
$d_{1}$ (nm)	1.2845	1.2803	1.2828	1.2784
$d_{2}$ (nm)	1.2947	1.2903	1.2929	1.2883
$\varepsilon_{HOMO}$	−0.13783	−0.14419	−0.14661	−0.15097
$\varepsilon_{LUMO}$	−0.12441	−0.12801	−0.13396	−0.13557
Gap	0.365	0.440	0.344	0.419
$E_{SCF}$	−7350.247114	−7342.011956	−7351.563136	−7343.186493

**Table 3:** Forbidden gap of ribbon C2 with and without polarization. The calculations were carried out with B3LYP/6-31G* and the geometry of the non-polarized AGNR. For the sake of comparison, results for ribbons without topological defects are also shown. All energies are in Hartrees, except the gap which is given in eV.

	7/9-AGRN		Without Defect 7/7		Without Defect 9/9	
	Non-Polarized	Polarized	Non-Polarized	Polarized	Non-Polarized	Polarized
$E_{B3LYP}$	−7350.24711	−7350.25368	−6435.41973	−6435.44606	−8266.25259	−8266.28214
$\varepsilon_{HOMO}$	−0.13783	−0.14645	−0.13097	−0.15643	−0.13254	−0.16135
$\varepsilon_{LUMO}$	−0.12441	−0.11571	−0.12859	−0.10275	−0.13032	−0.10104
Gap	0.365	0.836	0.065	1.460	0.060	1.640
$\Delta E_{Pol-NoPol}$	0.0066		0.0263		0.0295	

**Table 4:** Forbidden gap of 7/9-AGNR superlattice containing 1, 2, 4 and 8 unit cells (see Figure 1) as calculated by solving the PPP and Hu Hamiltonians within the unrestricted approximation. Calculations for polarized and non-polarized configurations were conducted. All results in eV.

	Gap (eV)				$E_{\mathrm{UHF}}$ (eV)			
	Non-Polarized		Polarised		Non-Polarized		Polarised	
$N_{c}$	PPP	Hu	PPP	Hu	PPP	Hu	PPP	Hu
C1	3.616	0.597	*	4.893	−1037.562	−894.092	*	−906.222
C2	2.085	0.173	3.035	4.841	−2078.726	−1792.47	−2079.175	−1815.905
C4	1.567	0.041	2.970	4.824	−4161.08	−3589.361	−4162.539	−3635.361
C8	0.088	0.003	3.069	4.819	−8092.643	−7183.256	−8095.469	−7273.997

* Non-existent.

## Data Availability

The data presented in this study are available on request from the corresponding author.
